# Endocrine evaluation of patients hospitalized with COVID-19 infection: A cross sectional analyses in tertiary level hospital

**DOI:** 10.12669/pjms.40.11.9340

**Published:** 2024-12

**Authors:** Tahir Ghaffar, Shaista Kanwal, Azizul Hasan Aamir, Niktash Khan Hadi

**Affiliations:** 1 Dr. Tahir Ghaffar, MBBS, FCPS (Med), FCPS Endocrinology, MRCP. Associate Professor, Department of Diabetes, Endocrinology and Metabolic Diseases, MTI Hayatabad Medical Complex, Peshawar, Pakistan; 2 Dr. Shaista Kanwal, MBBS, FCPS (Med), FCPS Endocrinology, MRCP. Assistant Professor Department of Diabetes, Endocrinology and Metabolic Diseases, MTI Hayatabad Medical Complex, Peshawar, Pakistan; 3 Dr. Azizul Hasan Aamir, MRCP, FRCP (Edin), FACE. Department of Diabetes, Endocrinology and Metabolic Diseases, MTI Hayatabad Medical Complex, Peshawar, Pakistan; 4 Dr. Niktash Khan Hadi, MBBS, FCPS Medicine, MRCP. Senior Instructor, Internal Medicine and Endocrinology, Shaukat Khanum Memorial Cancer Hospital and Research Center, Peshawar - Pakistan

**Keywords:** SARS COV-2, COVID 19, Thyroid Function Test, Serum Cortisol

## Abstract

**Objective:**

To evaluate common endocrine responses of the patients hospitalized with COVID 19 Infection at Hayatabad Medical Complex Peshawar.

**Methods:**

This was a prospective cross sectional study which included 66 patients having age 18 years and above with positive COVID 19 PCR, who were reported in COVID OPD for hospitalization in Isolation Units and Intensive Care Unit of Hayatabad Medical Complex Peshawar between June 15, 2020 to December 15, 2020. Patients with preexisting kidney or liver disease, those who had used steroids before enrollment and pregnant and lactating females were excluded. Patients were clinically assessed, and investigations were performed which included C- reactive protein (CRP), complete blood count (CBC), Covid PCR, thyroid function tests (TFTs), and cortisol levels.

**Results:**

The mean age of the study participants was 54.38±15.81 years. At baseline, 75%, 17%, and 3% patients had mild, moderate, and severe COVID-19, respectively. Thyroid Stimulating Hormones (TSH) levels were suppressed in 25% of patients, more significant in those with more severe infection. Raised cortisol levels were found in 94% of patients at admission without any prior use of steroids. As per clinical outcome is concerned, mortality occurred in 20% of patients while 80% recovered healthy.

**Conclusion:**

The findings of the results suggest an appropriate response of endocrine system to covid infection. The amount of cortisol and TSH changes were also associated with the severity of sickness.

## INTRODUCTION

COVID-19 is a viral disease caused by severe acute respiratory syndrome CORONA VIRUS-2 (SARSCoV2) infection. This lethal viral disease outbreak impacted over 36 million people worldwide in 2019 and led to the World Health Organization (WHO) declaring it a public health emergency and “pandemic”.^1^ This deadly virus affects all parts of the body and compromise their functions.^2^,^3^

The functional receptor for SARS-COV-2, angiotensin converting enzyme 2 (ACE2) is expressed in many endocrine glands.^4^ COVID 19 infection can cause thyroiditis either directly or indirectly (through immune dysregulation).^5^,^6^ It can mediate immune response that cause a cytokine storm by releasing proinflammatory cytokines.^7^ However, TFTs could be deranged due to non-thyroidal illness in sepsis. During all these events, free T3 (FT3) decreases due to inflammatory cytokines.

There is limited data in the literature on the effects of COVID-19 infection on the thyroid and adrenal glands.^8^ The hypothalamic pituitary adrenal axis response to systemic illness is variable and it also affects treatment in terms of need for corticosteroids. The Critical Illness-Related-Corticosteroid Insufficiency (CIRCI) entails reduced adrenal response to the extent of illness and inflammation^9^ in some patients while in others there is higher cortisol response. Tan et al. found higher plasma cortisol levels in COVID-19 patients and higher mortality compared to non-COVID-19 patients.^10^ One study reported the serious adrenal injury in the postmortem case series of patients infected with COVID-19. This study also stressed the need to monitor adrenal glands in acute infection and post recovery of the patients.^8^

Patients infected with SARS-COV2 if need hospitalization are referred to specialized isolation units. Being tertiary care hospital, having Intensive Care Units (ICU) along with isolated COVID clinics in this part of the country, patients with positive PCR for SARS-COV2 were referred from all over the province for evaluation and management. We designed this study to evaluate Thyroid and Adrenal functions of the patients hospitalized with COVID 19 Infection. The current study will improve the existing knowledge of physicians about endocrine involvement in COVID infected patients especially in the region.

## METHODS

This was a prospective study which comprised of 66 patients, age 18 years and above with COVID-19 infection. They had positive PCR and were referred for hospitalization in Isolation and Intensive Care Unit of Hayatabad Medical Complex Peshawar from June 15, 2020 to December 15, 2020. Symptoms like fever, dyspnea, cough, change in smell, taste and diarrhea were recorded and examination notes were also recorded like temperature, respiratory rate, pulse, blood pressure, and oxygen saturation. Serum cortisol, Thyroid Function Tests (TFTs), Complete Blood Count (CBC), Ferritin Levels and C Reactive Protein (CRP) values were recorded at admission. TSH, T4 and T3 and cortisol were assessed by the electrochemiluminescence assay and electro chemiluminescent Elecsys® respectively. CRP was measured by immunoturbidimetric assay Tina-quant®. The presence of pulmonary involvement was noted from X-rays and then High-resolution Computer Tomography scan (HRCT).

### Ethical Approval:

The study was conducted after approval of the Ethical Committee (Reference No. 1148, date: September 6, 2022).

Pregnant or lactating females, patients with prior kidney or liver diseases and patients who had used steroids before blood sampling were excluded. Based on the WHO criteria, mild infection group was the one who had signs and symptoms but no evidence of pneumonia. Patients with signs and symptoms but no severe pneumonia were considered moderate. Those having pneumonia and respiratory rate more than 30/min or SpO2<90% or severe respiratory distress were considered as having acute respiratory distress syndrome.^11^ Final outcome of the patient was also reordered as per discharge slip in terms of duration of stay, recovery and mortality.

For continues variables, mean ± SD and/or median (min-max) and for categorical variables percentages were calculated. For association of variables, the chi square test was used. For association of mortality with variables, a multivariate logistic regression model was used. The predictive model via stepwise selection using P = 0.05 as the entry value was used. Significance level was considered with p < 0.05. IBM SPSS version 26.0 was used for analysis.

## RESULTS

A total of 66 patients were included in the study. Most of the patients under study were 54 years of age (54.38±15.81) and 72% were male (Table-I). Most of the patients were admitted in Isolation unit (80%) while few required ICU admission (7%). Most of the patients had comorbidities (60%) that included Asthma 4%, Diabetes Mellitus 12%,

**Table-I:** Demographic baseline characteristic of COVID-19 Patients.

Variables	Patients (n=66)
***Age in Years***	
Mean ± S.D	54.38 ± 15.81
***Sex***	
Male	45 (68%)
Female	21 (32%)
***Duration since positive PCR test (days)***	
Median (Min-Max)	1 (0-12)
***Admitted in***	
Ward	12 (18%
Isolation	49 (75%)
ICU	5 (7%)
***Base Line COVID Severity***	
Mild	49 (75%)
Moderate	11 (17%)
Severe	6 (8%)
***Mean Average Length of Stay*** Mean ± SD	10.88 ± 12
***Clinical Out Come***	
Recovered	53 (80%)
Mortality Occurred	13 (20%)

Diabetes with Hypertension 16%, Diabetes with hypertension and Diabetic foot ulcer 14% and Hypertension 12%. At baseline, 75%, 17%, and 3% patients had mild, moderate, and severe infection respectively. Patients stayed admitted for 10 days in most cases with average length of stay (10.88 ± 12 days). As per outcome, mortality occurred in 20% of patients while 80% recovered healthy.

Common symptoms were fever, dyspnea, body aches and cough in 78%, 75%, 78% and 68% cases respectively. Change in smell, change in taste and diarrhea were also present in around 50% of cases. The radiological findings showed infiltrates on their chest x-ray in 68% cases.

Laboratory investigations revealed anemia (65%), leukocytosis (56%), lymphopenia (18%), and thrombocythemia (15%). Statistical analysis showed high neutrophil counts in 100%, Ferritin in 89%, and C reactive protein in 100% patients.

The endocrine system response in terms of thyroid function tests and cortisol levels is presented in Table-II. TSH levels were suppressed in 25% of patients and raised cortisol levels were found in 94% of patients. There was a significant reduction in TSH level with severity of infection with TSH of 0.85 in those with mild infection, 0.22 in those with moderate and 0.3 in those with severe infection (Table-III). Mean cortisol levels (42.81± 12.44) were high among 94% of the hospitalized patients, however those patients who recovered and those who died had mean cortisol of 40.92± 42 and 41±88 respectively.

**Table-II:** Distribution of Thyroid function test and Cortisol levels.

Levels	TSH (0.3-4.2)	Total T3 (0.8-2)	Total T4 (5.1-14.1)	Cortisol (5-18)
Elevated	3 (4%)	11 (16%)	19 (18%)	62 (94%)
Normal	47(71%)	49 (74%)	85 (80%)	
Low	16 (25%)	9 (10%)	6 (2%)	

**Table-III:** Severity of COVID Infection with Thyroid and Cortisol Levels.

COVID Grading	Mean TSH	Mean Total T3	Mean Total T4	Cortisol Levels	p Value
Mild	0.85±0.28	0.85±0.288	8.52±2.97	42.81±17.75	0.000
Moderate	0.22±0.44	2.48±1.84	16.42±4.30	48.92±18.26	0.000
Severe	0.14±0.12	4.82±2.44	18.44±4.44	52.48±20.24	0.000

Poor prognosis of the clinical outcome was observed among admitted patients. The multivariate logistic regression analysis revealed age, basal cortisol and presence of infiltration as significant on analysis.

According to the regression model, a one unit increase in age significantly increased hospital admission risk by 1.003 times (OR = 1.03, p = 0.029). Likewise, hospital admission risk was increased by 1.003 times with each unit increase in basal cortisol level (OR = 1.003, p = 0.024). A positive radiological finding significantly increased the hospital admission risk by three times (OR = 3.800, p = 0.026) (Table-IV). There was a significant association of radiological finding of the patients and clinical outcome during hospital stay (p value 0.00) among the admitted patients with SARS-COV2 infection.

**Table-IV:** Multiple logistic regression analysis to determine risk factors affecting admission.

Variable	B	SE	Exp (B)	p Value
Age	0.003	0.013	1.003	0.029
Cortisol Levels	0.062	0.013	1.003	0.024
Radiological Findings	1.38	0.65	3.800	0.026
Constant	-1.312	0.600	0.00	0.001

Multiple logistic regression analysis (backward LR); omnibus tests of model coefficient 0.001, Nagelkerke R square = 0.001; Hosmer and Lemeshow test = 0.105.

Multiple logistic regression analysis (backward LR); omnibus tests of model coefficient 0.001, Nagelkerke R square = 0.001; Hosmer and Lemeshow test = 0.105.

**Fig.1:**
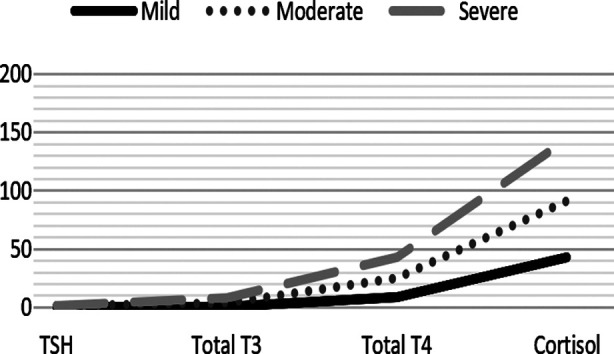
COVID severity vs TSH, Total T3, Total T4 and Cortisol.

**Fig.2:**
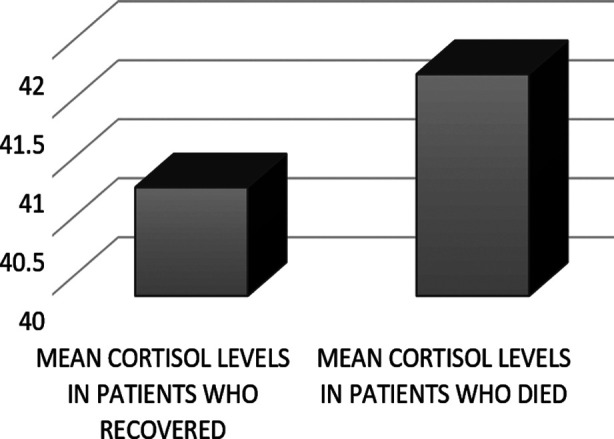
Bar Chart Shows Mean Cortisol Level Vs Clinical Outcome patients.

## DISCUSSION

Cortisol as stress hormone responds to emergency conditions / critical illness to prepare body for “fight or flight”.^12^ Higher levels of cortisol levels in the plasma in critically ill patient shows severity of the underlying disease, however inadequate response to stressors indicate HPA axis failure or adrenal insufficiency.^13^ These findings endorsed our study findings, where 94% of the SARS-COV 2 infected patients had raised plasma cortisol and it was associated with disease severity(p= 0.000). These findings are consistent with those investigated by Marpaung FR et al. where they revealed a significant correlation between Adrenocorticotropic hormone (ACTH) and cortisol in all SARS-COV2 infected patients within the survival group with p < 0:05 but not in the non- survived group.^14^ These results are like the findings of the study by Tan et al. where they determined that higher cortisol level was associated with the higher mortality rate of patients with COVID-19. Tan and colleagues found 42% increase in mortality with doubling of serum cortisol levels as severity marker of covid infection.^15^ A meta-analysis conducted to evaluate association between covid 19 severity and serum cortisol levels showed similar findings. This analysis found significantly high levels of cortisol in those with severe covid infection as compared to those with mild/moderate disease with standard mean difference of 1.48 mcg/dl (95% CI 0.51- 2.46).^16^ This shows consistency of finding across studies conducted looking at the association between two variables. The results of the current study showed that cortisol levels increased with the severity of SARS-COV2 infection. It was concluded that cortisol is better laboratory predictor of SARS-COV2 infection marker than CRP, D-Dimmer and Neutrophil to Lymphocyte ratio. The available literature shows association between cortisol levels as severity marker of covid infection that increases with positively with increasing severity of infection. There is no causes and effect relationship though covid may directly affect adrenals and cause reduction in cortisol contrary to the common scenario of stress induced hypercortisolism as this study has shown.

Non thyroidal illness is another manifestation of systemic response manifesting in thyroid hormones aberrancy without primary thyroidal illness. The most understood pattern of this condition is decrease in total and free T3 levels, low total T4 levels, and decreased TSH among 10% and higher among 5% of severely ill patients.^17^ In our study as the disease severity increased the TSH levels decreased to 0.14±0.12 (severe COVID group) but higher TSH was not observed in any case. A study conducted in India showed low TSH to be commonest abnormality in Covid patients though there was not any specific pattern as far as T3 and T4 are concerned.^18^ Most patients had normal T3 and T4 levels while elevated T3 and T4 were observed among 16% and 18% of the cases among which severely ill COVID patients had T3 and T4 4.82±2.44 and 18.44±4.44 respectively. The results of the current study are also supported by WHO report that revealed that patients who suffered severe acute respiratory syndrome (SARS) in many countries in 2002-2003 had deranged Thyroid function tests.^19^ A similar study but analyzing only Thyroid function in covid patients conclude similar findings. The more severe covid infection resulted in lower TSH and T3 levels reaching statistical significance with p < 0.001.^20^ A meta-analysis including 1237 patients and seven studies concludes heterogeneity in covid infection and TFTs results. The consistent findings reported were clinically severe covid infection and thyroid dysfunction.^21^

Chest X-ray findings are important in grading severity of Covid infection.^20^ Results of multiple logistic regression showed that risk factors for hospital admission of these severely ill patients were age, higher basal cortisol and infiltrates and pneumonic patches on the chest X-ray. A study by Salluh J et al.^19^ revealed higher mortality rate among pneumonia patients who had higher cortisol levels. Age was another poor prognostic factor along with chest x ray findings as predictor of mortality. A study aimed at renal outcomes in patients infected with Covid 19 also found increasing age as predictor of mortality.^21^-^23^

This study precludes evaluation of cortisol levels and TFTs in covid infection like in any infection. Both Cortisol and TSH may serve as marker of the disease severity especially in any new infection where specific markers are not known. Knowing severity of disruption of these two markers, physicians can better prepare for severe disease upfront resulting in better outcome.

### Limitations:

As the goal of the study was to assess thyroid and adrenal functions in hospitalized SARS-COV2 infection severity groups. Therefore, it was not possible to compare the diseased patients with the control group. The sample comprised of baseline investigation of the patients, although day-to-day variations in the course of treatment and the advancement of the disease could be observed.

## CONCLUSION

The most important feature of our study was evaluation of thyroid and adrenal functions and their correlation among SARS-COV2 infected hospitalized patients in terms of clinical outcome. The findings of the results were positive in relation to thyroid levels and cortisol levels among the severely ill patients. The effect of adrenal response should be kept in mind while treating COVID 19 infected patients. Low TSH was observed among 25% of the patients and was the most common abnormality among kind of alteration of thyroid functions.

### Author’s Contribution:

**TG:** Conceived, designed, did literature review, performed statistical analysis & drafted the manuscript.

**SK:** Data collection, data analysis, literature review and interpretation of data, and helped in drafting.

**AHA:** Conceived, designed, and critically revised the manuscript.

**NKH:** Data collection, did literature review and interpretation of data.

All authors provided final approval for publication of the manuscript and are responsible for the integrity of the study.
